# Yield and In Vitro Antioxidant Potential of Essential Oil from *Aerva javanica* (Burm. f.) Juss. ex Schul. Flower with Special Emphasis on Seasonal Changes

**DOI:** 10.3390/plants10122618

**Published:** 2021-11-29

**Authors:** Suzan Marwan Shahin, Abdul Jaleel, Mohammed Abdul Muhsen Alyafei

**Affiliations:** 1 Department of Integrative Agriculture, College of Agriculture and Veterinary Medicine, United Arab Emirates University, Al Ain 15551, United Arab Emirates; drsuzan.s@uaqu.ac.ae (S.M.S.); abdul.jaleel@uaeu.ac.ae (A.J.); 2 Research and Development Head, Umm Al Quwain University, Umm Al Quwain 536, United Arab Emirates

**Keywords:** *Aerva javanica*, sandy soil, hydrodistillation, antioxidant activity, seasonal variation, GC-MS, angustione, trichomes

## Abstract

The essential oil (EO) of the desert cotton (*Aerva javanica* (Burm. f.) Juss. ex Schul.) was extracted by hydrodistillation, from *A*. *javanica* flowers growing in the sandy soils of the United Arab Emirates (UAE) wild desert. The influence of seasonal variation on flowers’ EO yield was studied. The flowers’ EO yield obtained from spring samples (0.011%) was significantly the highest followed by early summer (0.009%), winter (0.007%), and autumn samples (0.006%), respectively. The flowers’ EO antioxidant analysis were tested by DPPH, FRAP and ABTS assays (in vitro). Results proved that *A. javanica* flowers’ EO, isolated during the four seasons, is a good source of natural bioactive antioxidants. Based on the three tested assays, the highest antioxidant activity was recorded in the spring. Testing of the chemical composition of the flowers’ EO was conducted for the season with the highest yield and the best antioxidant performance, recorded in spring, by a combination of gas chromatograph (GC) and gas chromatograph-mass spectrometer (GC-MS). This led to the identification of 29 volatile components, in which the flowers’ oil was characterized by angustione as a major compound. Photos by scanning electron microscope (SEM) showed prominent availability of star-shaped trichomes in the epidermis of the flowers.

## 1. Introduction

*Aerva javanica* (Burm. f.) Juss. ex Schul. (English names: desert cotton, snow bush) (Arabic names: Al ara’, twaim, efhe, tirf) [1], a perennial xerophyte belonging to the Amaranthaceae family (common name: cockscomb). This shrub grows up to 100 cm. It has a woody base, erect and branched stems, covered with fine hairs. The leaves are alternate with greyish-green color on very short stalks, lance-shaped to oblong (1–1.5 × 4–5 cm) with clear veins and midrib on the underside covered with hairs. The flowers are aromatic, soft like cotton and generally available throughout the year [2]. It has five petals on a long spike (5–10 cm) from the leaf nodes. The open flowers are white and the buds are pinkish. The bracts (leaf-like structure) just below the flowers are covered with long woolly white hairs that become more intense as the season progresses. The inside of the fruits have a woolly covering, including one small black or brown shiny seed (0.1 × 0.15 cm) [1]. 

The genus *Aerva* is well-known as an important medicinal genus, including many species with proven biological activities, such as, antioxidant, hypoglycemic, analgesic, antivenin, antimalarial, and anthelmintic activities [2,3]. *A. javanica* is an important medicinal and commercial plant. It is recommended by Ayurvedic medicine as one of the best sources of natural remedies (e.g., to treat the bladder and kidney stones) [3]. *A. javanica* is an essential oil-bearing shrub (or small tree), common in tropical and subtropical dry areas, with various folk applications related to its flowers in traditional herbal practices. For example, the flowers were mixed with water as a paste to stop wounds bleeding, and to pack suppurating wounds after cleaning. Furthermore, flowers were used for curing kidney and rheumatism problems [1,2].

Consequently, it is of great interest to investigate the flowers’ EO yield, which is the first objective of this work, in order to recommend the best harvesting season with the highest yield. Furthermore, for the sake of testing the quality, the antioxidant activity of *A. javanica* flowers’ EO will be tested for the first time (in vitro) and during the four seasons, which is the second objective of this research, seeking for a scientific justification for the rich ethnomedicinal applications of this plant’s flowers. Besides, the volatiles of flowers’ EO will be identified, to the best of our knowledge, for the first time in the literature. The same would be identified for the best flowers’ EO yield obtained. In addition, the microscopic photos under scanning electron microscope are included in this study; providing a better understanding of the finer morphology of the trichomes of the flower epidermis.

## 2. Materials and Methods

### 2.1. Plant Material Identification

The mature plants of *A. javanica* were identified by Ali El-Keblawy, Professor of Plant Ecology (College of Sciences, University of Sharjah) and Tamer Mahmoud, Researcher Botanist (Sharjah Seed Bank and Herbarium, Sharjah Research Academy). Furthermore, fresh *A. javanica* flowers were collected from the study location in Al Ain on 5th of November (temperature: 22–33 °C) and identified by Mohamed Taher Mousa from the Biology Department of the UAEU; the voucher specimen of the plant was deposited in the Herbarium of the Biology Department, UAEU (voucher No. 14668).

### 2.2. Study Location and Plant Collection Schedules

The natural communities of *A. javanica* were growing wild in the Al Ain area (latitude: 24°11′ N, longitude: 55°39′ E). The mature flowers of *A. javanica* were randomly collected and separated in the early morning according to the following time schedule and details:

The flowers were harvested once at around the middle of each season, except for the summer batch of flowers, which was collected on the first of June instead of July. The same was done, due to the unavailability of flowers, after the first of June; since they will both complete and end their life cycles. The collected flower batch permitted examining the seasonal variation influence on the flowers’ EO yields. The details of the harvesting schedules are as follows:

Spring: Mid-April (21–35 °C, average: 28 °C).

Summer: First of June (23–47.2 °C, average: 35.1 °C)

Autumn: End of October (24–35 °C, average: 29.5 °C).

Winter: Mid-January (11–22 °C, average: 16.5 °C).

All weather data were obtained from the iPhone application, “Weather”, which collects data from the Weather Channel: Aqbiyah Weather Station, UAE.

### 2.3. Soil Physical and Chemical Analysis

The analysis of soil physical properties (done in triplicate) showed that the soil has a sandy soil texture, measured by dry sieve method (sand% “2 to 0.053 mm”: 97.30 ± 1.02; silt% “0.032 mm”: 2.09 ± 1.01; clay% “<0.032 mm”: 0.61 ± 0.37). 

The analysis of soil chemical properties (done in triplicate) showed that the soil electric conductivity (EC) is 0.971 ± 0.064 mS measured by EC meter with pH 7.6 ± 0.035 measured at 21 °C by pH meter; both tests were done for disturbed samples. The soil samples consisted of 0.520 ± 0.047% organic matter measured by the Walkley–Black method. The utilized soil consisted of 0.0761 ± 0.015% nitrogen, 6.074 ± 0.021 ppm phosphorus and 102.54 ± 0.468 ppm potassium. The total nitrogen was measured by Vario MACRO cube CHNS and manufactured by Elementar Co. (Langenselbold, Germany), while the total phosphorus and potassium were measured by inductively coupled plasma atomic emission spectroscopy (ICP-OES), model 710-ES. The total soil calcium carbonate (CaCO_3_) was 27.841 ± 0.844% measured by calcimeter method.

### 2.4. Statistics and Experimental Design

The data were subjected to a statistical analysis using SPSS statistical software version 21. One-way analysis of variance (ANOVA) followed by Tukey (honestly significant differences, HSD) multiple range test were employed, and the differences between the individual means were deemed to be significant at *p* ≤ 0.05 significance level. 

The figures and tables illustrate the significant differences between the individual groups by letters, in which the use of either different letters in the same treatment group, the use of the symbol (*), or both, mean that there is a significant difference at *p* ≤ 0.05 (unless a different significance level is specified), while the use of similar letters or absence of letters means that the difference between the same individual group is not significant. 

The experimental design of all the conducted experiments is completely randomized. The number of samples and their replications used are illustrated separately in the description of each experiment.

### 2.5. EO Extraction by Hydrodistillation

The EO isolation was done by hydrodistillation method using Clevenger type apparatus. The seasonal variation influence on flowers’ EO yields were examined, in which three (3) replicates were considered. 

The seasonal variations’ influence on *A. javanica* flowers’ EO was tested. The summary of the tested treatments (mentioned as points) is represented in Table 1. 

#### 2.5.1. Sample Preparation 

The flowers collected throughout the year in each of the four seasons (as described in Table 1) were washed thoroughly with distilled water three times, and left in a well ventilate shaded area (27 °C) for two weeks (until completely dried). The flowers were then weighed (dry weight), labelled, and stored in clean and closed paper bags until use for further analysis. 

On the distillation day, the flowers were cut into homogenous sizes (2 mm), measured by U.S. metric sieves (SOILTEST, ASTM), and directly subjected to hydrodistillation. It is worth mentioning that the size of *A. javanica* flowers, which is 2 mm, was obtained by separating the flowers from the mother plant by hand. The dry matter contents were measured using the following formula:

$$\mathrm{DM}\left(\%\right)=\frac{\mathrm{DW}}{\mathrm{FW}}\times 100$$

where DM is the dry matter, FW is the fresh weight, and DW is the dry weight.

#### 2.5.2. Extraction Conditions and Yield Determination

The hydrodistillation process was performed for around 100 g of plant matrix (dry weight) at 85 °C, for a period of 4 h, until no further EO was extracted. At the end of the distillation, the EO was accumulated as a waxy extract collected and easily separated from water. Thus, drying with anhydrous sodium sulfate (anhydrous Na_2_SO_4_) was not needed. The pure collected EO was weighed and stored in a sealed glass amber vial at 4 °C until analyzed. After the distillation, the Clevenger apparatus was subjected to a process using water, soap, methanol, and ethanol. 

The EO yield was calculated based on the plant dry weight and the herbal extract weight using the following equation:

$$\mathrm{EO}\;\mathrm{yield}\;\left(\%\right)=\frac{W_{\mathrm{EO}}}{W_{\mathrm{plant}}}\times 100$$

where, W_plant_ is the dry weight of the plant (in grams) and W_EO_ is the weight of the extracted EO (in grams).

### 2.6. Antioxidant Activity

The antioxidant activity was determined in microplate to evaluate the antioxidant activity (in vitro) by spectrophotometer (Microplate reader, BioTek, EPOCH 2) using a 96-well plate. The tested antioxidant assays were: DPPH (2,2-diphenyl-1-picryl hydrazyl), FRAP (ferric reducing antioxidant power), and ABTS (2, 2-azinobis (3-ethylbenzothiazoline-6-sulfonate)). 

#### 2.6.1. Sample Preparation 

EO waxy samples of *A. javanica* (flowers) for different seasons (in triplicate) were dissolved in 500 μL of dimethyl sulfoxide (DMSO) and vortexed very well.

#### 2.6.2. Standard Curves 

**Figure 1:**
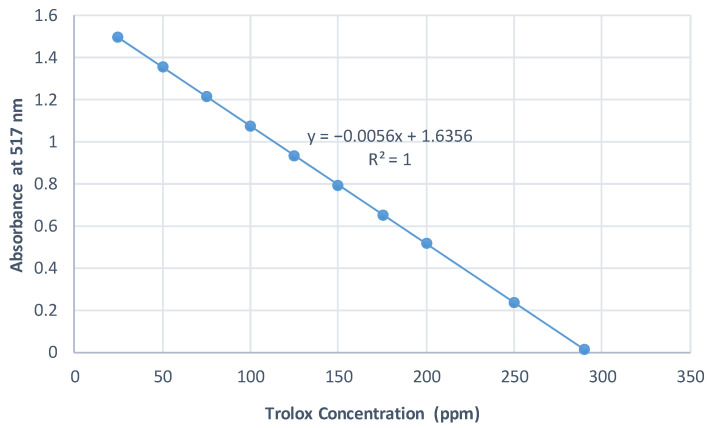
Standard curve of DPPH assay.

**Figure 2:**
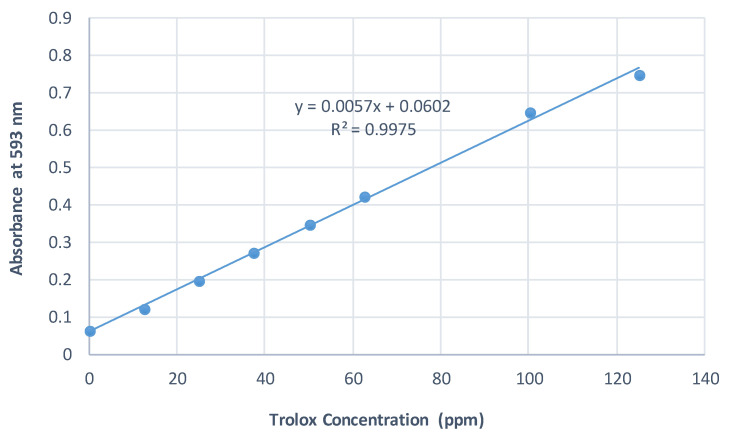
Standard curve of FRAP assay.

**Figure 3:**
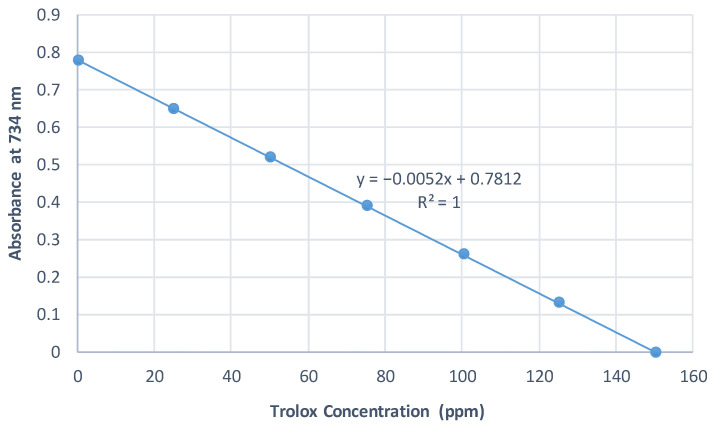
Standard curve of ABTS assay.

The standard curves were prepared using Trolox as a strong antioxidant reagent. The absorbance of Trolox at different concentrations was measured at 517 nm, 593 nm, and 734 nm for DPPH, FRAP, and ABTS assays, respectively. The results of all conducted assays were calculated and expressed as mg Trolox equivalents/g EO extract. The standard curves are illustrated in Figure 1, Figure 2 and Figure 3.

### 2.7. Chromatographic Analysis 

EO sample analysis was performed on an Agilent gas chromatograph (GC) equipped with flame ionization detection (FID) and an HP-5MS capillary column, 30 m length × 0.25 mm internal diameter, 0.25 μm film thickness, and temperature programmed as follows: 70–300 °C at 10 °C/min. The carrier gas was helium at a flow of 3.0 mL/min; injector port and detector temperature were 250 °C and 300 °C, respectively. One microliter of the sample (diluted with acetone, 1:10 ratio) was injected by splitting and the split ratio was 1:100.

#### GC-MS Analysis

The gas chromatography-mass spectrometry (GC-MS) analysis was performed on an Agilent C 5975 GC-MS system with an HP-5MS capillary column (30 m × 0.25 mm internal diameter × 0.25 μm film thickness). The operating conditions were the same conditions as described above for the chromatographic analysis. The mass spectra were taken at 70 eV. The scan mass range was from 40 to 700 m/z at a sampling rate of 1.0 scan/s. The quantitative data were obtained from the electronic integration of the FID peak areas.

The components of the EO were identified by their retention time (RT), retention indices (RI) and mass spectra, relative to C9–C28 n-alkanes, computer matching with the library of NIST Mass Spectrometry Data Center (NIST11.L), as well as by comparison of their retention indices with those of the authentic samples or with the data already available in the literature [4]. The percentage of composition of the identified compounds was computed from the GC peak areas without any correction factors and was calculated relatively.

### 2.8. Plant Morphology by SEM Photos

The finer morphological details of the *A. javanica* flower (collected from the experimental location in mid-April) were examined using a scanning electron microscope (SEM) XL Series, Netherlands. The process was done at the Electron Microscope Unit, College of Medicine, UAEU.

#### 2.8.1. Chemicals

Chemicals used were as follows: fixative, phosphate buffer, osmium tetroxide buffer, and ethanol.

#### 2.8.2. Sample Preparation Protocol

First, the specimens were cut into 4 × 4 mm pieces, and transferred into fixative at pH 7.2 in a 7 mL glass vial with attached lids and stand overnight at 4 °C. The next day, specimens were washed in 0.1 M phosphate buffer (3 times, 20 min each), and stored at 4 °C. Furthermore, 1 mL of buffered 1% osmium tetroxide was then added to each vial, and mixed for 1 h at room temperature on a Rota mixer. After that, the osmium tetroxide was removed and samples were washed three times in distilled water (for 2 min each). The samples were dehydrated through a series of ethanol washes. The next day, the specimens were washed again (twice) with 100% ethanol. The final flower sample was kept inside a desiccator for four days to allow gradual drying.

## 3. Results and Discussion

### 3.1. EO Extraction by Hydrodistillation

EO physical characteristics: The flowers’ EO has a yellowish color with waxy nature, and has a characteristic floral odor similar to the fresh flowers.

### 3.2. Effect of Seasonal Variation

#### 3.2.1. Quantitative Analysis (by Yield)

**Figure 4:**
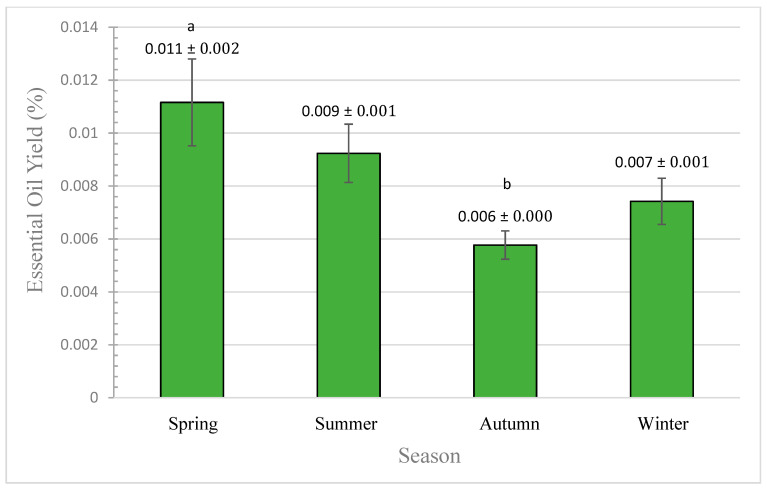
Effect of seasonal variation on flowers of *A. javanica* EO yield. The different letters are indicators of the significant variation between groups.

The influence of seasonal variations on the EO yield (%, *v*/*w* of dry weight) of *A. javanica* air-dried flowers is illustrated in Figure 4. The mean results are represented with their standard deviations (SDs) and standard deviation error bars.

The analysis of variance, by one-way ANOVA, showed significant effect for the seasonal variation on flowers’ EO yield (quantitatively) at 0.042 significance level (*p* ≤ 0.05). The analysis of multiple comparisons between groups by Tukey HSD test showed significant variations of EO yield obtained in spring at 0.036 significance level with results obtained in autumn. Our results are similar to other studies found in the literature, which report that a seasonal variation has significant influence on EO yield [5,6,7]. According to Hussain et al. [5], the highest EO yield was obtained in spring, which was reduced afterward. Therefore, our findings are in agreement with their obtained results.

Annually, *A. javanica*’s flowering stage started approximately in November (end of autumn season), continuing to grow in the winter, reaching maturity in the spring, and completing the life cycle by the beginning of summer. This corresponds with the trend of our obtained EO yields, which shows the lowest results in the autumn, followed by an increased EO yield in winter, reaching the maximum yield in spring, which then starts decreasing by the beginning of summer.

Our results are in agreement with the results obtained by Hussain et al. [5], Omer et al. [6], and Villa-Ruano et al. [7], which report that the seasonal variation has a significant influence on the quantitative EO yield. According to Hussain et al. [5], high EO yield was recorded in spring and reduced afterward, which is similar to our results.

During spring, the flowers reach the maturity stage, thus the EO yield from the glandular cells will reach maximum levels, which will play a major role in the pollination process. However, with the arrival of the summer, which is characterized by high temperature and high variations between daily minimum and maximum temperatures, the proline content of the leaves will reach maximum levels, while flowers will end their life cycle, and accordingly the glandular cells will collapse, thus flowers’ EO content will be reduced.

Based on our results, the best season to extract the highest quantitative EO yield (0.011 ± 0.002%) obtained from *A. javanica* flowers is spring, followed by early summer (0.009 ± 0.001%) and winter (0.007 ± 0.000%). While extracting the oil from flowers collected during autumn provides the lowest yield (0.006 ± 0.001%), thus not recommended.

Our results are similar to other studies found in the literature, which report that seasonal variation has a significant influence on the quantitative EO yield [5,6,7].

#### 3.2.2. Qualitative Analysis (by Antioxidant Activity)

##### DPPH Assay

The DPPH molecule (2,2-diphenyl-1-picryl hydrazyl) is one of the few stable organic nitrogen radicals. It is characterized by deep purple color, with maximum absorbance at 517 nm wavelength. The antioxidants cause the reduction reactions against the free radical DPPH by pairing; it has an odd electron with the free radical scavenging antioxidant. This reaction causes color loss of the purple DPPH due to the formation of the reduced DPPH-H. The higher the color loss, the higher the antioxidant concentration would be, thus higher DPPH scavenging activity [8].

In this work, the antioxidant concentration was calculated as Trolox equivalent from a calibration curve and the antioxidant activity was expressed as mg Trolox eq/g of extract.

**Figure 5:**
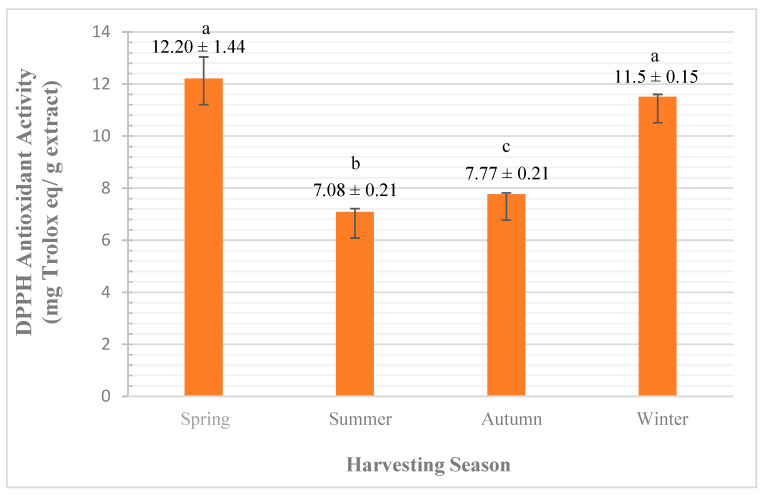
Effect of seasonal variation on *A. javanica* EO by DPPH assay. The different letters are indicators of the significant variation between groups.

The analysis of variance, by one-way ANOVA, showed a significant difference (at <0.0005 significance level) for the influence of seasonal variation on the antioxidant activity of *A. javanica* flowers’ EO using DPPH assay. As shown in Figure 5, the highest antioxidant activities are obtained in the spring (12.20 ± 1.44 mg Aq/g), followed by winter (11.5 ± 0.15 mg Aq/g), autumn (7.77 ± 0.21 mg Aq/g), and summer (7.08 ± 0.21 mg Aq/g), respectively.

The analysis of multiple comparisons between groups by Tukey HSD test showed significant variations of antioxidant activity recorded in spring with summer and autumn both at <0.0005 significance level, while no significant variation between spring and winter was observed. The antioxidant activity recorded in winter was significant with summer and autumn also at <0.0005 and 0.001 significance levels, respectively.

Although, there are many studies that link the influence of exposing the plant to biotic and abiotic stress factors with the high productivity of EO [9,10,11]. However, in our work there was a negative correlation between the severity of seasonal impacts and the EO yield, which is in agreement with the results obtained by Hussain et al. [5], who reported that during the year the highest antioxidant activity of basil EO was recorded in spring, while the lowest activity was recorded in summer.

Linking the quantitative and qualitative results of *A. javanica* EO obtained from flowers to our current antioxidant DPPH results shows that the high antioxidant activity recorded in the spring, which was then reduced significantly by the arrival of summer, could be due to the variation in amounts of major compounds, which causes the reduction in the availability of the electron-donation group.

It is worth mentioning that the *A. javanica* flowering stage starts approximately in November (end of autumn) and continues growing into the winter reaching maturity in spring, and completing the life cycle by the beginning of summer. This supported the trend of our obtained antioxidant results by DPPH assay.

Our results are similar to other studies found in the literature, which report that the seasonal variation has a significant influence on the qualitative EO yield [5,6,7]. According to Hussain et al. [5], basil EO extracted in spring was rich in oxygenated monoterpenes, while the oil obtained in summer was rich in sesquiterpene hydrocarbons. Meaning that, seasonal variation has a significant influence on the EO qualitative yield, which is similar to our obtained antioxidant results.

##### FRAP Assay

**Figure 6:**
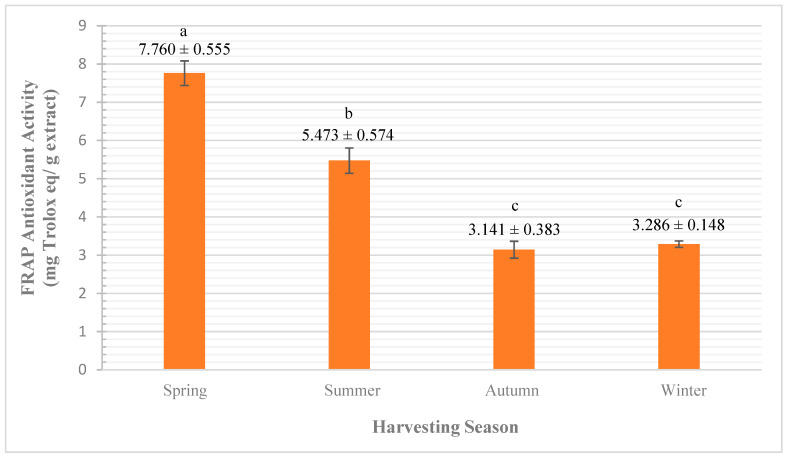
Effect of seasonal variation on *A. javanica* EO by FRAP assay. The different letters are indicators of the significant variation between groups.

The analysis of variance, by one-way ANOVA, showed a significant difference (at <0.0005 significance level) for the influence of the seasonal variation on the antioxidant activity of *A. javanica* flowers’ EO using FRAP assay. As shown in Figure 6, the highest antioxidant activities were obtained in the spring (7.76 ± 0.55 mg Aq/g), followed by summer (5.47 ± 0.57 mg Aq/g), winter (3.28 ± 0.148 mg Aq/g), and autumn (3.14 ± 0.38 mg Aq/g), respectively.

The analysis of multiple comparisons between groups by Tukey HSD test showed significant variations of antioxidant activity recorded in spring with the antioxidant results recorded in summer at 0.001 significance level. Furthermore, the variation between the results of spring with autumn and winter were significant both at <0.0005 significance level. While no significant variation between autumn and winter was observed.

Although, there are many studies that link the influence of exposing the plant to biotic and abiotic stress factors with the high productivity of EO [9,10,11]. However, in our work there was a negative correlation between the severity of seasonal impacts and the EO yield, which is in agreement with the results obtained by Hussain et al. [5], who reported that during the year the highest antioxidant activity of basil EO was recorded in spring, which reduced afterward.

Our results are in agreement with the results obtained by Hussain et al. [5], who reported that during the year the highest antioxidant activity of basil EO was recorded in spring, which reduced afterward.

In general, our results are similar to other studies found in the literature, which report that the seasonal variation has a significant influence on the qualitative EO yield [5,6,7].

It is worth mentioning that the *A. javanica* flowering stage starts approximately in November (end of autumn) and continues growing in the winter reaching maturity in the spring, and completing the life cycle by the beginning of summer. This supports the trend of our obtained antioxidant results by FRAP assay.

In general, the antioxidant activity obtained from FRAP assay is due to the availability of hydrogen donating ability, which can be linked to the existence of adjacent substituted groups (e.g., hydroxyl).

##### ABTS Assay

**Figure 7:**
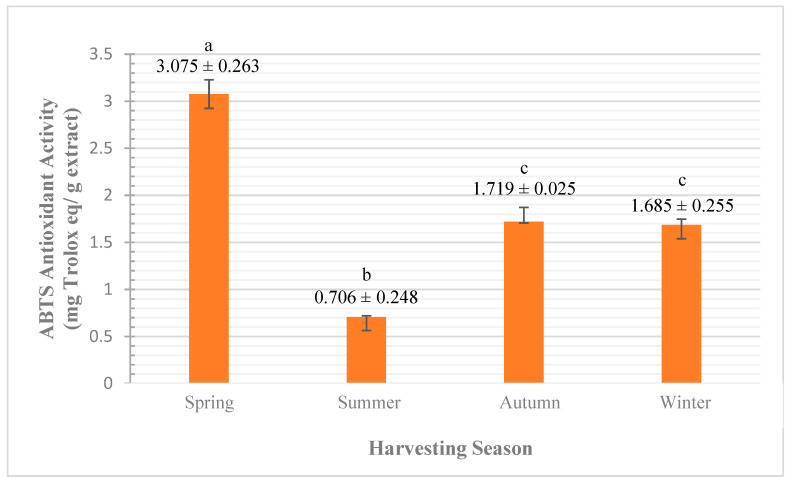
Effect of seasonal variation on *A. javanica* EO by ABTS assay. The different letters are indicators of the significant variation between groups.

The analysis of variance, by one-way ANOVA, showed a significant difference at <0.0005 significance level for the influence of seasonal variation on the antioxidant activity of *A. javanica* flowers’ EO using ABTS assay. As shown in Figure 7, the highest antioxidant activities obtained in the spring (3.075 ± 0.263 mg Aq/g) are followed by autumn (1.719 ± 0.025 mg Aq/g), winter (1.685 ± 0.255 mg Aq/g), and summer (0.706 ± 0.248 mg Aq/g), respectively.

The analysis of multiple comparisons between groups by Tukey HSD test showed significant variations of antioxidant activity recorded in spring with antioxidant results of all seasons at <0.0005 significance level. Furthermore, the variation between the results of summer with spring, autumn, and winter were significant at <0.0005, 0.002, and 0.003 significance levels, respectively. While no significant variation between autumn and winter was observed.

Still, there are many studies that link the influence of exposing the plant to biotic and abiotic stress factors with high production of EO [9,10]. However, in our work there was a negative correlation between the severity of seasonal impacts and the EO yield, which is in agreement with the results obtained by Hussain et al. [5], who reported that during the year the highest antioxidant activity of basil EO was recorded in spring, while the lowest activity was recorded in summer.

Linking the quantitative results of *A. javanica* EO obtained from flowers to our current antioxidant ABTS results shows that the high antioxidant activity recorded in the spring, which then reduced significantly by the arrival of the summer, could be due to the variation in the amounts of the major compounds, which causes the reduction in the availability of the electron-donation group.

It is worth mentioning that the *A. javanica* flowering stage started approximately in November (end of autumn) and continued growing into winter, reaching maturity in the spring, and completing the life cycle by the beginning of summer. This supports the trend of our obtained antioxidant results by ABTS assay.

Our results are similar to other studies found in the literature, which report that the seasonal variation has a significant influence on the qualitative EO yield [5,6,7].

##### Correlation between Antioxidant Assays

The correlation analysis using Pearson’s correlation test was done to evaluate the strength of the relationship between the results of antioxidant activity obtained by applying the three antioxidant assays: DPPH, FRAP, and ABTS. The Pearson’s correlation coefficients were calculated using SPSS statistical software (version 21) at ≤0.01 significance level, in which the significant effect will be illustrated in this section’s tables using the sign (*).

Our correlation analysis of the flowers’ EO antioxidant activity measured for the four seasons is shown in Table 2, Table 3, Table 4 and Table 5. The illustrated correlation coefficients of spring flowers show strong positive relationship between all tested antioxidant assays. The correlation coefficient of spring flowers was found to be strong (at ≤0.01 significance level) between DPPH and FRAP, DPPH with ABTS, and FRAP with ABTS. While, the correlation coefficient measured for the remaining seasons shows a very strong positive relationship (significant at ≤0.01 significance level) between all the tested antioxidant assays.

### 3.3. Chemical Composition (by GC-MS)

Referring to our previous flowers’ EO results (yield and antioxidant analysis), it was shown that the highest yield with the best antioxidant activity of the oil was recorded in spring, in which the chemical composition of the oil was investigated in this section.

**Figure 8:**
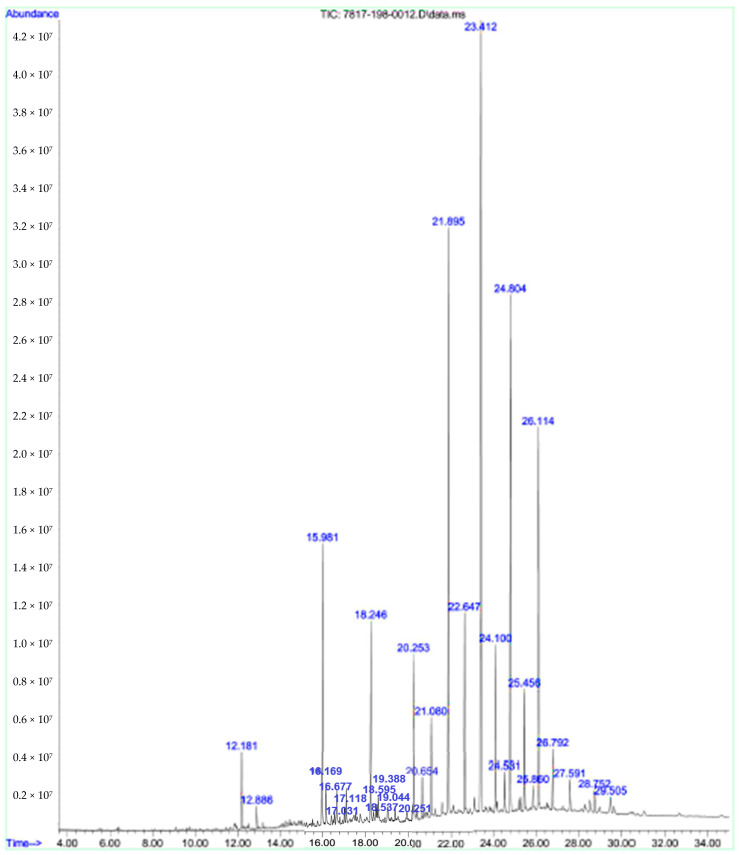
The chromatogram of *A. javanica* air-dried flowers’ EO for spring.

The chromatogram result of *A. javanica* air-dried flowers’ EO extracted during the spring is illustrated in Figure 8. The complete chemical composition of EO, retention time (RT) in minutes, retention indices (RI), and percentages (%) of identified compounds of the flowers’ oil are all represented in Table 6.

The GC-MS analysis of the flowers’ EO showed the chemical composition of the oil with 29 identified volatiles (representing 100% of the oil). The major compounds were angustione (20.72%), evodone (14.77%), *methyl*-cresol acetate (12.49%), *γ*-elemene (9.15%), and verbenone (6.06%). Furthermore, 4-hydroxybenzaldehyde (4.56%), (*4Z*)-decen-1-ol (4.07%), and 1-tetradecene (3.98%) were identified as minor components.

Our results show that the *A. javanica* flowers’ EO is a rich source of potential hydrocarbons, and supporting the results published by Samejo et al. [12] and Samejo et al. [13]. Our results are justifying the rich ethnomedicinal applications of the flowers [14,15]. Angustione is a ketone reported to have antioxidant, anticancer, and antiviral (anti-HIV) biological activity [16]. Therefore, spring is more favorable to be considered as a collection season of *A. javanica* flowers that provides EO with a higher percentage of angustione, and consequently, better biological activity.

It is worth mentioning that verbenone is a monoterpene (bicyclic ketone terpene) that has a pleasant characteristic aroma, thus it is used in a wide range of industries (e.g., herbal remedies, herbal teas, spices, aromatherapy, and perfumery) [17]. Furthermore, verbenone is an insect pheromone, thus used to control insects [18]. Similarly, *γ*-elemene is a sesquiterpene that has a floral aroma and reported to cause antifungal and antioxidant activity [19].

Truly, the chemical composition of the EO would be significantly varied according to the harvesting season [5,6,7]. Therefore, the decision of deciding the favorable harvesting season depends upon the proposed application of the extracted oil.; meaning that different applications can consider different harvesting seasons accordingly.

### 3.4. Morphology by SEM

**Figure 9:**
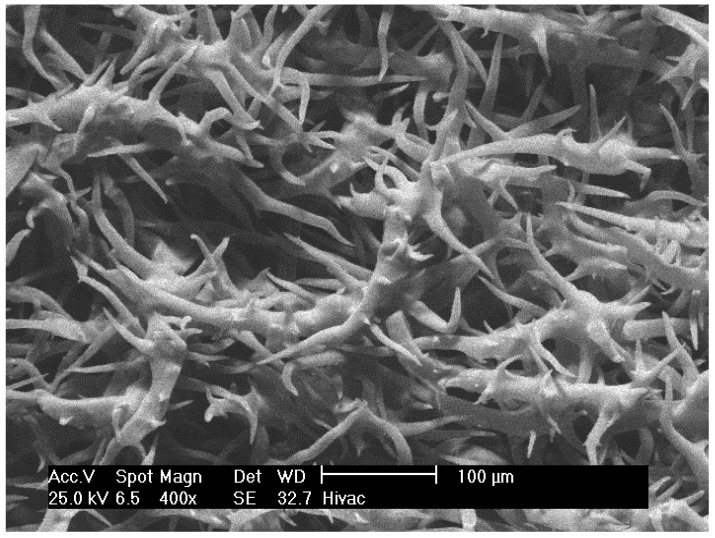
Flower epidermis trichomes of *A javanica* (magnification: 400×).

**Figure 10:**
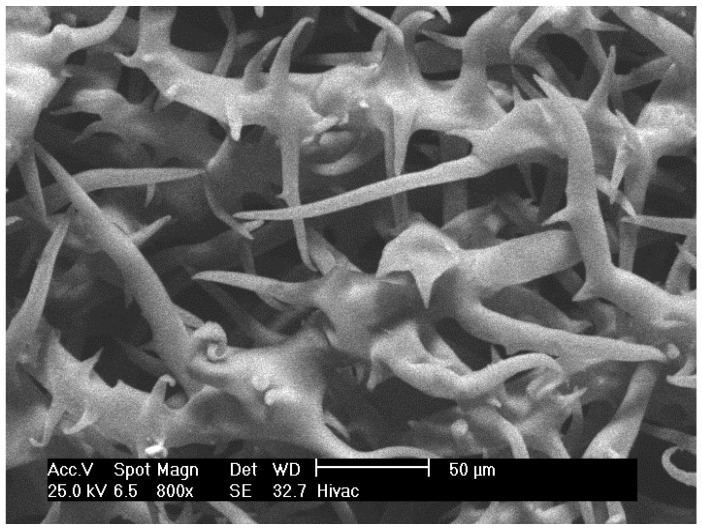
Flowers epidermis trichomes of *A javanica* (magnification: 800×).

SEM photos for the flower’s epidermis are illustrated (Figure 9 and Figure 10), in which the photos show pubescent indumentum, consisting of stellate (star-shaped) base cells with uniseriate tapered ending. The trichomes are prominent and appear at high density. The SEM photos for the flowers are illustrated for the first time in the literature.

The glandular trichomes indicating thero-tolerance by dissipating heat from the surface of the plant, which play a major role in enhancing the water use efficiency through adjusting the osmotic potential, which is a natural adaptation mechanism for desert plants in arid regions. Besides, the glandular trichomes are suspended to produce secondary metabolites, such as EOs, and acting as defense phytochemicals against biotic (e.g., herbivores) and abiotic (e.g., high temperature, low precipitation) stress factors [11,20].

The appearance of all the mentioned morphological features justifies the fact that *A. javanica* is a desert xerophytic plant that can survive high temperatures and high sun exposure rates with minimal water requirements. Furthermore, the existence of glandular trichomes on the epidermis of the flower supports the fact that *A. javanica* is an EO-bearing plant.

## 4. Conclusions

*Aerva javanica* is a xerophytic EO-bearing plant. The seasonal variation showed a significant effect on the flowers’ EO yield, in which the best season to extract the highest EO yield is spring (0.011%). Similarly, the seasonal variation has a significant effect on the antioxidant activity of *A. javanica* flowers’ EO, showing the best activity during the spring, tested in vitro using antioxidant assays (DPPH, FRAP, and ABTS).

The chemical composition of the flowers’ EO extracted in the optimal season (during spring) is characterized with 29 identified volatiles, of which the major volatiles are angustione (20.72%) and evodone (14.77%).

*A. javanica* flowers’ EO is a good resource of natural antioxidants, and has a high potential for the pharmaceuticals industry. Future clinical antioxidant studies for fractionate EO compounds are highly recommended.

## Figures and Tables

**Table 1:** Summary of *A. javanica* experimental treatments.

Plant Part	Drying Methods	Particle Size	Harvesting Season
Flowers	Air-Drying: Shaded at 25 °C	2 mm	Spring (Mid-April) Summer (First of June) Autumn (End of October) Winter (Mid-January)

**Table 2:** Pearson correlation coefficients of antioxidant activity of spring flowers.

	DPPH	FRAP	ABTS
DPPH	NA	+0.933	+0.994
FRAP	+0.933	NA	+0.968
ABTS	+0.994	+0.968	NA

(NA) means Not Applicable.

**Table 3:** Pearson correlation coefficients of antioxidant activity of summer flowers.

Antioxidant Assays	DPPH	FRAP	ABTS
DPPH	NA	+1 *	+1 *
FRAP	+1 *	NA	+1 *
ABTS	+1 *	+1 *	NA

(*) means significant at ≤0.01 significance level. (NA) means not applicable.

**Table 4:** Pearson correlation coefficients of antioxidant activity of autumn flowers.

Antioxidant Assays	DPPH	FRAP	ABTS
DPPH	NA	+1 *	+1 *
FRAP	+1 *	NA	+1 *
ABTS	+1 *	+1 *	NA

(*) means significant at ≤0.01 significance level. (NA) means not applicable.

**Table 5:** Pearson correlation coefficients of antioxidant activity of winter flowers.

Antioxidant Assays	DPPH	FRAP	ABTS
DPPH	NA	+1 *	+1 *
FRAP	+1 *	NA	+1 *
ABTS	+1 *	+1 *	NA

(*) means significant at ≤0.01 significance level. (NA) means not applicable.

**Table 6:** Composition of *A. javanica* EO obtained from air-dried flowers during spring.

No.	Compound	RT	RI	Percent Composition (%) Spring
1	*2E*,*4E*-Octadienol	12.184	1113	1.35
2	*cis*-Limonene oxide	12.883	1132	0.42
3	Verbenone	15.973	1204	6.06
4	(*2E*)-Octenol acetate	16.171	1208	1.14
5	endo-Fenchyl acetate	16.573	1218	-----
6	*Methyl*-(2E)-nonenoate	16.678	1221	1.20
7	*nor*-Davanone	17.033	1228	0.39
8	Thymol methyl ether	17.115	1132	0.84
9	*tetrahydro*-Linalool acetate	17.121	1231	-----
10	Pulegone	17.261	1233	-----
11	Carvotanacetone	17.768	1244	-----
12	(*4Z*)-Decen-1-ol	18.246	1255	4.07
13	(*2E*)-Decenal	18.485	1260	0.44
14	*cis*-Chrysanthenyl acetate	18.537	1261	0.43
15	Geranial	18.595	1264	0.97
16	*trans*-Carvone oxide	19.044	1273	0.71
17	*(2Z)*-Hexenyl valerate	19.388	1282	0.39
18	*n*-Tridecane	20.245	1300	3.79
19	Isoamyl bemzyl ether	20.653	1310	0.95
20	*2E*,*4E*-Decadienol	21.078	1319	2.08
21	3-oxo-ρ-Menth-1-en-7-al	21.580	1330	-----
22	Evodone	21.877	1137	14.77
23	4-Hydroxybenzaldehyde	22.641	1355	4.56
24	Angustione	23.392	1372	20.72
25	(*E*)-Methyl cinnamate	23.556	1376	-----
26	1-Tetradecene	24.092	1388	3.98
27	α-Chamipinene	24.448	1396	-----
28	Cyperene	24.529	1398	1.16
29	*methyl*-Cresol acetate	24.791	1403	12.49
30	(*2E*,*4E*)-Undecadienal	25.281	1415	-----
31	(*E*)-Trimenal	25.450	1419	2.77
32	*cis*-Thujopsene	25.858	1429	0.80
33	*γ*-Elemene	26.103	1434	9.15
34	α-Humulene	26.791	1452	1.65
35	(*2E*)-Dodecen-1-ol	27.589	1469	1.05
36	α-Selinene	28.755	1498	0.88
37	Menthyl isovalerate	29.507	1516	0.80
